# Research on multi-cluster green persimmon detection method based on improved Faster RCNN

**DOI:** 10.3389/fpls.2023.1177114

**Published:** 2023-06-06

**Authors:** Yangyang Liu, Huimin Ren, Zhi Zhang, Fansheng Men, Pengyang Zhang, Delin Wu, Ruizhuo Feng

**Affiliations:** ^1^ School of Engineering, Anhui Agricultural University, Hefei, Anhui, China; ^2^ School of Mechanical Engineering, Yangzhou University, Yangzhou, China

**Keywords:** multi-cluster green persimmon recognition, occlusion images, DetNet, attention mechanism, multi-scale feature fusion

## Abstract

To address the problem of accurate recognition and localization of multiple clusters of green persimmons with similar color to the background under natural environment, this study proposes a multi-cluster green persimmon identification method based on improved Faster RCNN was proposed by using the self-built green persimmon dataset. The feature extractor DetNet is used as the backbone feature extraction network, and the model detection attention is focused on the target object itself by adding the weighted ECA channel attention mechanism to the three effective feature layers in the backbone, and the detection accuracy of the algorithm is improved. By maximizing the pooling of the lower layer features with the added attention mechanism, the high and low dimensions and magnitudes are made the same. The processed feature layers are combined with multi-scale features using a serial layer-hopping connection structure to enhance the robustness of feature information, effectively copes with the problem of target detection of objects with obscured near scenery in complex environments and accelerates the detection speed through feature complementarity between different feature layers. In this study, the K-means clustering algorithm is used to group and anchor the bounding boxes so that they converge to the actual bounding boxes, The average mean accuracy (mAP) of the improved Faster RCNN model reaches 98.4%, which was 11.8% higher than that of traditional Faster RCNN model, which also increases the accuracy of object detection during regression prediction. and the average detection time of a single image is improved by 0.54s. The algorithm is significantly improved in terms of accuracy and speed, which provides a basis for green fruit growth state monitoring and intelligent yield estimation in real scenarios.

## 1 Introduction

An orchard is a complex ecosystem that occupies an important position in rural economic development. Digitalization, information technology, and intelligent management technology of orchards can collect fruit growth information in real time, quickly, and objectively, which will serve as reference and a basis for the scale of orchard production (Ingram and Maye, 2020; Maheswari et al., 2021). China is one of the world’s major producing countries of persimmons (Zaman et al., 2022), Persimmons grow in orchards with complex and changeable ecological environments, and orchards are mostly grown on small plots, mainly relying on manual labor. In different seasons of orchard environment, work will be subject to certain restrictions, with the characteristics of high labor intensity, low efficiency, high cost and short cycle. It can be seen that mechanization has shown a high degree of importance in the development of small plot planting, and the demand for machine substitution is becoming more and more urgent. Efficient identification and precise positioning of persimmons is the key to achieving intelligent operation in orchards (Fu et al., 2020), and the key to achieving intelligent operation in orchards is the accurate identification and positioning of persimmons. However, because the color of green persimmon epidermis is extremely close to the color of environmental branches and leaves, the influence of light variation, shooting angle, and branch shading in the unstructured environment of the forest poses significant challenges to the detection of green persimmon targets and restricts the development of intelligent operation technology in orchards.

Artificial intelligence technology has advanced the development of intelligent and informative agriculture (Kawamura et al., 2022). Convolutional neural networks (CNN) have emerged as a popular tool for fruit target detection with powerful self-learning ability to extract rich abstract visual features and stronger robustness to light, fruit overlap, and occlusion (Lin et al., 2018; Afonso et al., 2020). The accurate and efficient identification of fruit targets with similar background colors in complex environments plays an important role in agricultural production links such as intelligent picking, growth monitoring, and fruit detection (Jia et al., 2022b). Wei et al. (2022) proposed a two-stage D2D detection framework to detect fruits such as green persimmons in the orchard environment, and the test showed that the model had the best detection effect, but the model detection did not take into account the factor that the fruits were occluded in the natural environment. Zhang et al. (2020a) used a fast fruit detection model based on Faster-RCNN for the shortcomings of conventional detection methods that make it challenging to meet real-time requirements in the detection process, which can achieve real-time fruit detection, but did not consider the influence conditions of various factors in complex environments. Peng et al. (2018) proposed an improved SSD deep learning fruit detection model with replacement of the backbone feature extraction network, which can address the current issue of low fruit recognition rates in natural environments. However, the model improves the target recognition accuracy but still lacks in speed. The improved FCO network proposed by Long et al. (2021) increases the accuracy of apple target detection from multiple angles. However, the model assigns targets to be detected at different scales to different network layers for prediction, which increases the computational effort to some extent. Fu et al. (2018) proposed a deep learning model based on LeNet CNN for multi-cluster kiwifruit image recognition to achieve fast and accurate recognition of multi-cluster kiwifruit fruits under field conditions. Although LeNet improves the accuracy and speed of kiwifruit recognition, it does not address the issues of false or missed recognitions due to branch and leaf occlusion or overlapping. Yue et al. (2019) studied apple detection by adding a boundary-weighted loss function to an improved Mask RCNN network, which led to more accurate boundary detection results. However, the network was tested under natural conditions without considering the small target problem. A method for recognizing apples based on R-FCN was presented by Wang and He (2019) to address the issue that it is challenging to identify pre-thinning apples under natural conditions due to various factors. Although the recognition of pre-thinning apple targets, which is challenging to achieve by conventional approaches, can also be widely used in the recognition of other small targets with similar background colors, this technique does not look into the various target scales present in the same image. Nevertheless, this approach does not examine the influence of different target scales within a single image. Jia et al. (2022a) used the two-stage Mask rcnn segmentation algorithm to accurately segment green fruits such as persimmons in the natural environment, although the algorithm has high detection accuracy for green obscured fruits, but does not comprehensively consider the unclear recognition of fruit boundaries caused by lighting in the natural environment.

Although the above research can identify fruits, there are still some drawbacks including a high computational cost, a poor recognition accuracy, a single environmental factor, and a lack of research on multiple clusters of green fruits in complex backgrounds. At present, convolutional neural networks have more mature applications not only in handwritten character recognition (Soujanya et al., 2022; Singh and Chaturvedi, 2023) and vehicle detection (Chen and Li, 2022; Gomaa et al., 2022), but also in the recognition of fruits such as apples (Hu et al., 2021; Liu et al., 2021), pears (Li et al., 2022) and oranges (Ren and Zhu, 2021), but there is no relevant literature on the use of neural networks for green persimmons recognition, it seriously restricts the development of intelligent persimmon orchard operation robots. In this study, a multi-cluster green persimmon recognition algorithm based on improved Faster RCNN was designed by improving the backbone feature extraction network. The algorithm’s connection structure is combined with multi-scale features to achieve efficient recognition and accurate localization of multi-cluster green persimmons in complex background environments. The improved algorithm not only keeps the model’s parameters from being drastically increased, but it also enhances feature propagation and improves the speed while ensuring the accuracy, which provides the basis for the development of smart orchard recognition technology.

## 2 Multi-cluster green persimmon recognition network design for complex natural environments

### 2.1 Faster RCNN network structure

In this study, the Faster RCNN model for multi-cluster green persimmon detection is developed based on the PyTorch framework. Faster RCNN is a typical representative of two-stage detection models (Ren et al., 2017). It is a feature detection network composed of two neural networks combining the region generating network RPN and Fast RCNN network models.

The RPN is a lightweight region generation module that produces a better “Proposal” of the proposed frame. It uses a sliding window to slide over the feature map generated by the pre-trained network model, producing a one-dimensional vector for each sliding position, which is then convolved with a 3 × 3 convolution kernel to further extract the frame features. The multi-task binary classification loss function of RPN is represented as follows:


(1) $$L(\left\{p_{i}\right\},\left\{t_{i}\right\})=\frac{1}{N_{cls}}\sum_{i}L_{cls}(p_{i},p_{i}^{*})+\lambda \frac{1}{N_{\text{reg}}}\sum_{i}p_{i}^{*}L_{\text{reg}}(t_{i},t_{i}^{*})$$

where the classification loss function is:


(2) $$Lcls=-[p_{i}^{*}\text{log}(p_{i})+(1-p_{i}^{*})\text{log}(1-p_{i})]$$

with *p_i_* being the probability that the *i*-th anchor is predicted to be the target; *p_i_* is 1 for positive samples and 0 for negative samples. The bounding box regression loss function is:


(3) $$L_{\text{reg}}(t_{i},t_{i}^{*})=\sum_{i}smooth_{L_{i}}(t_{i}-t_{i}^{*})$$

(4) $$\mathrm{smooth}L_{i}(x)=\{\begin{array}{c} 0.5x^{2}\text{if}\left|x\right|<1\\ \left|x\right|-0.5\;\text{otherwise}\; \end{array}$$

where *t_i_* is the regression parameter of the bounding box of the *i*-th anchor and $t_{i}^{*}$ is the regression parameter of the real box corresponding to the *i*-th anchor.

The Fast RCNN algorithm uses the selective search (ss) algorithm to generate 1k-2k candidate regions in an image, and the entire image is then input into the CNN to obtain the corresponding feature maps. The ss algorithm projects the candidate regions generated on the original image onto the feature map to obtain the corresponding feature matrices, and each feature matrix is finally scaled to a uniform size by ROI Pooling layer, followed by spreading the feature map through a series of fully connected layers to predict the class to which the target belongs and the bounding box regression parameters. The loss function of Fast RCNN can be expressed as:


(5) $$L(p,u,t^{u},v)=L_{cls}(p,u)+\lambda \left[u\geq 1\right]L_{\text{loc}}(t^{u},v)$$

where *p* is the SoftMax probability distribution predicted by the classifier, *u* the real class label of the target, *t^u^* represents the regression parameters of the corresponding class *u* predicted by the bounding box regressor, and *v* represents the bounding box regression parameters of the real target.

**Figure 1:**
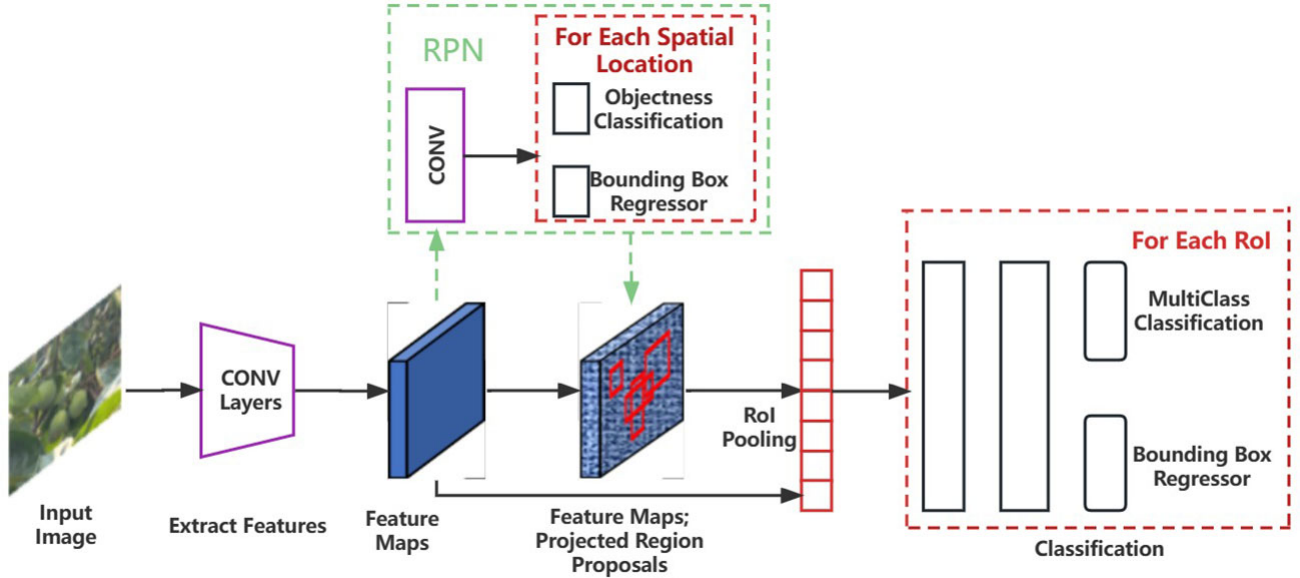
Architecture of Faster RCNN model.

In this study, the joint training method of RPN Loss and Fast RCNN Loss is used to train the network by backpropagating the losses of these two parts. The architecture of the Faster RCNN network is shown in **Figure 1**, and the workflow can be divided into three steps: (1) the features of the input image are extracted through the pre-training network; (2) the extracted features are passed through the RPN model to produce a certain number of candidate frames; (3) the predicted classification and regression results are input to ROI Pooling with both candidate frames and image features to classify the candidate regions, determine their categories, and fine-tune their positions.

### 2.2 Improved feature extraction network structure based on DetNet backbone network

Target detection requires not only identifying the class of objects, but also spatially locating their bounding box. Most conventional backbone networks use classical convolutional neural network models, such as ResNet (Zhang et al., 2020b), AlexNet (Li and He, 2018) and VGGl6 (Ren et al., 2022), as well as other classification models, but their feature map’s spatial resolution is too small, which is not conducive to the localization of large and small targets. DetNet-59 (Subramanian et al., 2022), which has strong target detection performance, is used in this study as the backbone network for feature extraction, and the null convolution and residual structure are used to prevent the objects and small targets in the complex background of the images from disappearing while ensuring that the obtained feature maps have clear boundaries. The DetNet-59 model keeps the original ResNet-50 stages 1 to 4 unchanged while stages 5 and 6 are newly introduced. As a result, the high spatial resolution of the feature map is maintained, and a large perceptual field is obtained. while also avoiding the multiple up sampling of FPN to achieve a better detection effect.

**Figure 2:**
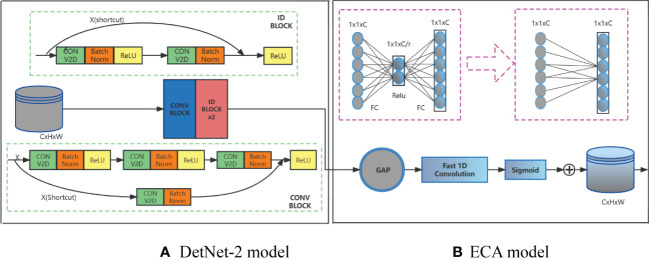
Structure of DetNet-2 imposed attention mechanism. **(A)** DetNet-2 model. **(B)** ECA model.


In their natural environment, green persimmons often experience fruit overlap issues as well as branch and leaf occlusion, which reduces the model detection accuracy. In this study, the channel attention module (ECA) is introduced in the DetNet network structure to enhance the extracted feature representation capability and high resolution of the feature map, as shown in **Figure 2**. ECA (Wang et al., 2020) is an extremely lightweight channel attention module that does not require dimensionality reduction to achieve local cross-channel interaction and avoids the effect of dimensionality reduction on the channel attention learning. The feature map is compressed and a 1×1×C feature map is obtained. Dynamic convolution is used to learn different channel features for the compressed feature map, and a one-dimensional convolution kernel size adaptive selection method is used to select the number of convolution kernel channel neighbors *k* in order to determine the coverage of local cross-channel interactions. The channel-attentive feature map 1×1×C is then combined with the original input feature map H×W×C by channel-by-channel multiplication for channel attention, and the feature map with channel attention is output. This attention mechanism significantly reduces the model’s complexity while performance is maintained. To avoid cross-validation, the value of *k* needs to be optimized. This value can be adaptively determined as a function of the total number of channels C:

(6) $$\kappa =\varphi (∁)={\left|\frac{\log_{2}∁+1}{2}\right|}_{odd}$$

where κ is the number of neighbors per channel, *C* is the total number of channels, and |*χ*|_odd_ represents the nearest odd number.

The feature utilization of the three convolutional blocks of shallow (DetNet-2), middle (DetNet-4) and deep (DetNet-6) layers is enhanced by the ECA mechanism to focus on the relevant information corresponding to the specific dimension extracted in the channel during the three attention calculations. The attention distribution is first computed over all input information. To select the color, texture, and semantic feature information corresponding to each of the three attention mechanisms from several different feature dimensions, one type of relevant feature information is introduced for each, and the correlation between the input image feature dimensions and the relatively introduced feature information is calculated by the scoring function:


(7) $$\alpha_{r}=softmax(s(h_{r},q))$$

where *a* is the attention distribution, *h* is the feature dimension of the input image, *q* represents the specific feature information parameters, and:


(8) $$s(h,q)=v^{T}tanh(w_{x}+u_{q})$$

where *s* is the scoring function, *w*,*u*, and *v–* are learnable feature network parameters, and *r* represents channel location parameters.

The weighted average of the input information is then computed based on the attention distribution. The soft attention mechanism is used to obtain the results after the query, and the weighted average of the feature dimension information across multiple channels in the image is obtained for each channel of the feature map to determine the Attention value:


(9) $$att(h,q)=\sum_{r=1}^{C}\alpha_{r}h_{r}$$

where *C* is the total number of channels and *r* is the channel position parameter.

After three weightings, the channel attention mechanism is used to enhance the target attention faster and directly, which improves the dense target detection as well as the anti-background interference ability. To improve the recognition accuracy of multiple clusters of green persimmons in natural environments, high weights are assigned to increase the attention to detail information such as texture and edges of green persimmons, as well as to overlapping and occluded targets that are more difficult to recognize. whereas, low weights are assigned to suppress useless information.

### 2.3 DetNet-based multi-scale feature fusion network

**Figure 3:**
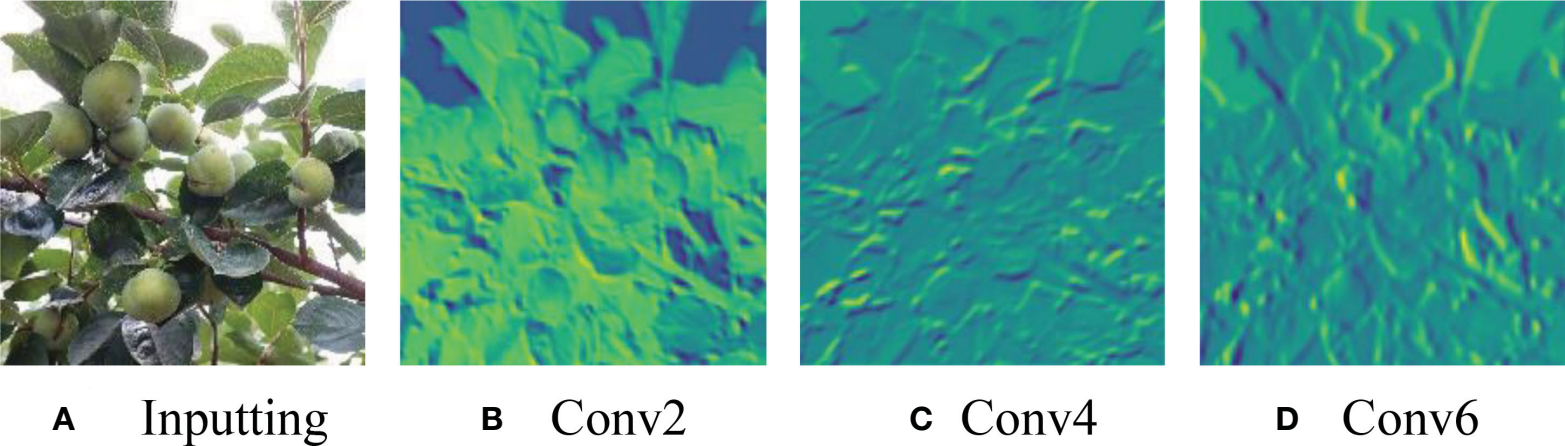
Feature visualizations of the original image **(A)** and three different depth convolution layers **(B–D)** in DetNet.

The backbone in Faster-RCNN is used to extract the target features through layer-by-layer abstraction, and the RPN performs feature extraction on the last layer of convolutional response obtained by the underlying CNN. Convolutional neural networks have higher resolution in the process of feature extraction with shallow layer features and more accurate target locations. In this study, the shallow layer tends to extract persimmon color features, and with the increase in convolutional layers, further features such as persimmon edge shape and texture are extracted. The deep layer features have stronger semantic information after multiple convolution operations and can more effectively convey the image information. This study extracts more abstract features in the green persimmon image in the deep layer and ignores the more detailed information in the image. The results of feature visualization in DetNet with different convolutional layers are shown in **Figure 3**.

The amount of feature information that can be gleaned from an image is constrained when the target is set against a complex background that is comparable to it and is affected by various lighting conditions. Feature extraction from only one layer cannot adequately detect and localize the target object. Therefore, this study uses a serial layer-hopping connection structure to combine features from several abstraction levels, and then trains a predictor on the combined features to achieve DetNet-based multi-scale feature fusion. To use multiscale features generated by multiple scale network layers, the dimensionality (feature map size and number of channels) and the magnitude must be considered. In this study, the shallow DetNet-2 layer’s features are maximally pooled to make them consistent with the scale of the middle and deep layer feature maps. The maximum pooling formula for its output feature map is:


(10) $$H_{out}=\frac{H_{in}+2\times \text{padding}\left[0\right]-\text{dilation}\left[0\right]\times \left(\text{kernel\_size}\left[0\right]-1\right)-1}{\text{stride}\left[0\right]}+1$$

(11) $$W_{out}=\frac{W_{in}+2\times \text{padding}\left[1\right]-\text{dilation}\left[1\right]\times \left(\text{kernel\_size}\left[1\right]-1\right)-1}{\text{stride}\left[1\right]}+1$$

where, kernel_size is the max pooling window size, stride is the step size for moving the max pooling window, padding is the number of layers of zeros appended to the input, and dilation is a parameter that controls the step of elements in the window.

**Figure 4:**
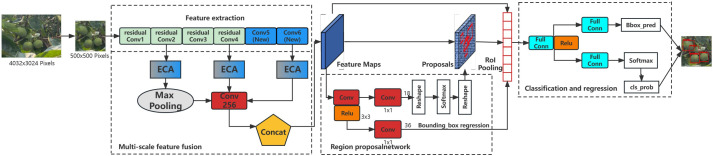
Schematic diagram of improved Faster RCNN-based multi-cluster green persimmon recognition algorithm structure.

Maximum pooling can obtain local information and better preserve the features on the texture while reducing the feature map size. The size of the feature map output from DetNet-2 is transformed to 14 × 14 with 256 channels by maximum pooling. The three feature vectors with 256 channels of the feature map are then fused early to form the candidate box region features to detect green persimmon in complex environments. The improved Faster RCNN multi-cluster green persimmon recognition algorithm is shown in **Figure 4**, which increases the sensitivity of the model to green persimmon features by enhancing the attention mechanism, and increases the resolution of feature mapping by fusing multiple layers of deep, medium, and shallow image features to achieve the effect of complementary advantages, thus improving the detection accuracy.

### 2.4 Improvement of region suggestion network based on K-means clustering

The anchor frame scheme in Faster RCNN is established for the PASCALVOC 2012 dataset, and the aspect ratio of anchor frames includes three ratios of 1:2, 1:1 and 2:1 and three sizes, so each object has 9 fixed ratios of anchor frames, and the default anchor frame ratios cannot be accurately identified due to the different aspect ratios of different datasets. Therefore, this work uses the K-means clustering algorithm to determine the anchor frame generation scheme, and groups the sizes of the real frames in the training samples to determine the most appropriate *a priori* frame sizes to improve the overlap rate between the predicted bounding boxes and the actual targets, thereby improving the algorithm’s detection performance and hastening its convergence.

Anchor box generation method: First, nine samples are randomly selected as the initial cluster centers, and the Euclidean distance is computed between each sample and the center of each cluster. Each sample is then assigned to the cluster center closest to it and the cluster centers are updated. The Euclidean distance is calculated as follows:


(12) $$d(X_{i},C_{k})=\sqrt{{(x_{0}^{i}-C_{0}^{k})}^{2}+{(x_{1}^{i}-C_{1}^{k})}^{2}+\ldots +{(x_{n}^{i}-C_{n}^{k})}^{2}}$$

where, *X_i_* is the number of samples in the dataset and *C^k^* is the set of initial cluster centers.

**Figure 5:**
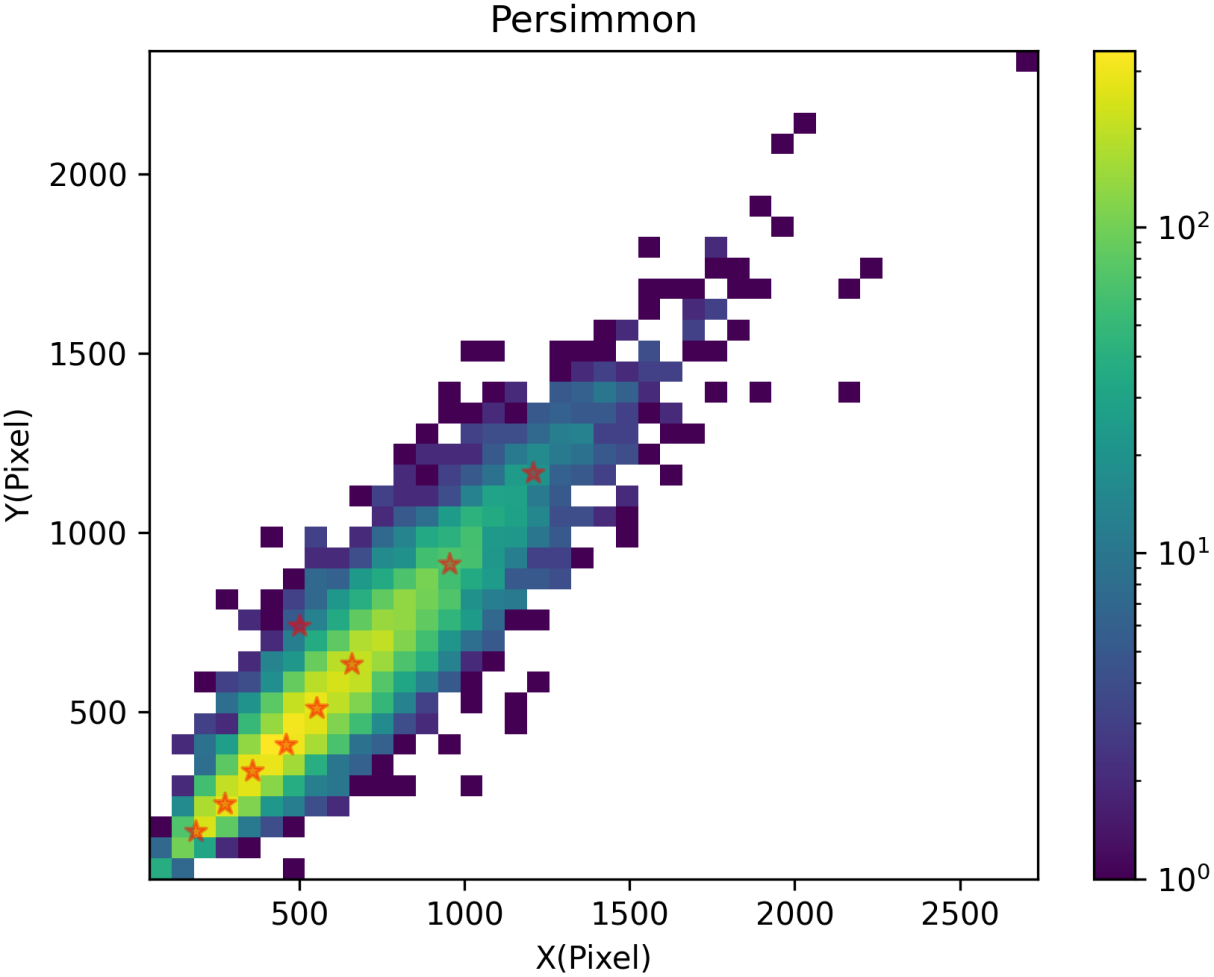
Graph of clustering results.

The mean value of all samples in each cluster is then calculated as the new cluster center until the cluster center no longer changes or the cluster center changes very little to satisfy a given termination condition. The anchor frames suitable for the dataset are regenerated by clustering, and the proportions and size of the nine anchor frames are obtained by training. The generated clustering results are shown in **Figure 5**.

## 3 Materials and methods

### 3.1 Image collection

Persimmon images were collected at the National Persimmon Germplasm Resource Nursery in Hefei, Anhui Province, China, from July 10 to August 31, 2022. The experimental sample database consists of 9300 images collected under natural light, with different light intensities at multiple angles and distances for various scenarios, including single fruit, multiple fruit, branch leaf shading, and multiple clusters of fruit overlapping, with an image resolution of 4608 × 3456. The shooting distance is within the range of 10cm~50cm, the number of persimmon fruits included in a single picture is about 2~12, and the number of persimmon fruits included in a single picture is about 12~40 when the shooting distance is greater than 50cm.

**Figure 6:**
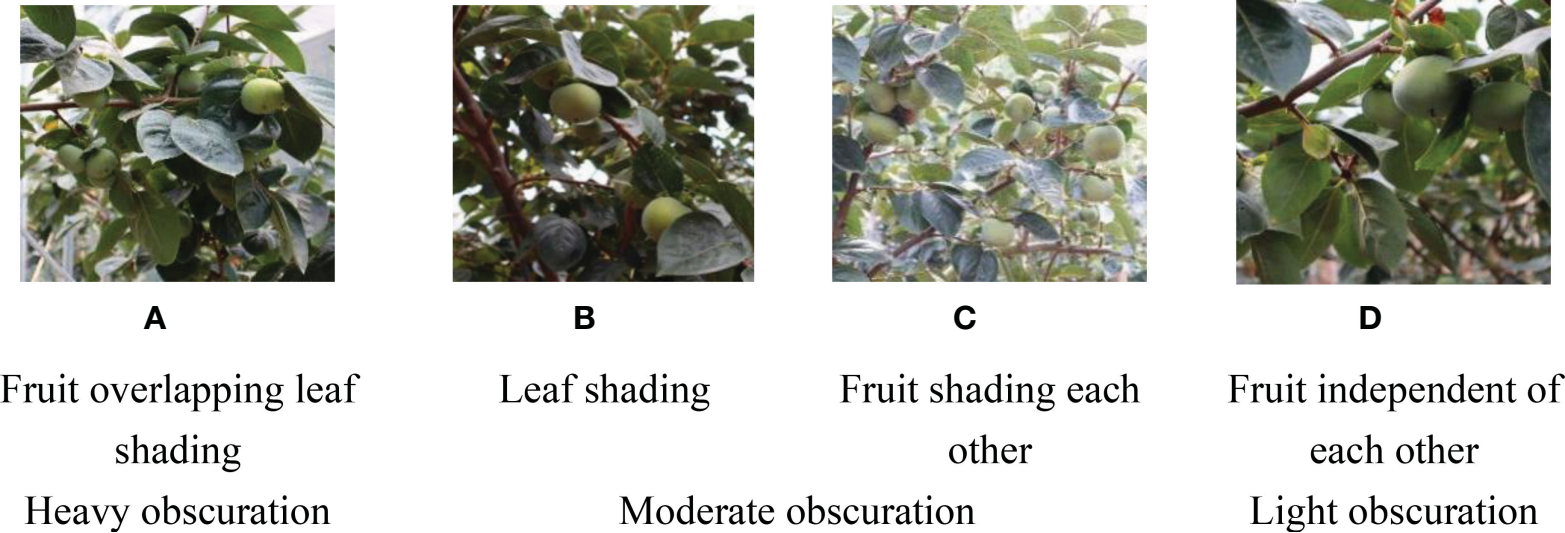
Examples of persimmon images with different occlusion degree **(A–D)**.

The quality of the dataset determines the quality of model training and the accuracy of predictions. In the early process of identification and detection of green persimmons, the factor of fruit occlusion seriously affects the accuracy of model recognition, and the data set is divided into three levels according to the severity of persimmon fruit occlusion, which improves the complexity of the dataset and the generalization ability of the model, so as to facilitate the detailed and accurate test results, which is more conducive to the analysis and solution of subsequent problems. As shown in **Figure 6**, and the statistical results are shown in **Table 1**. There are 3069 images with heavy obscuration, and the target in the image is obscured to 60%~80%; 3255 images with moderate obscuration, and the target is obscured to 30%~60%; 2976 images with light obscuration, and the target is obscured to less than 30%.

**Table 1:** Number of persimmons and images with different shading degrees.

Obstruction degree	Number of images/frame	Target obscured degree/%
Heavy obscuration	3069	60~80
Moderate obscuration	3255	30~60
Light obscuration	2976	0~30
Total	9300	≤80

### 3.2 Experimental environment configuration

The specific operating environment parameters of this study are shown in **Table 2**. The comprehensive sample data were divided into training and testing sets according to the ratio of 9:1, the sample labels were produced using PASCAL VOC dataset format, and the targets with more than 80% occlusion in the images were ignored in the labeling process. The results of clustering by k-means were [21,19], [33,28], [44,40], [55,50], [67,62], [78,74], [94,89], [111,108], and [143,36]. The number of epochs was set to 500, the learning rate Lr was set to 0.01, the optimizer was the stochastic gradient descent (SGD), the momentum was set to 0.9, and the intersection over union (IoU) was set to 0.5. TensorBoard was used to record the data during the training process, the training loss and learning rate changes were logged for each iteration and the weights were saved.

**Table 2:** Operating environment parameters.

Hardware	Configuration	Environment	Version
CPU	Intel Core i7-12700	Python	3.7.13
GPU	RTX 3060	PyTorch	1.7.1+cu110
RAM	64 G	CUDA	11.0
Hard disk	520 G	CUDNN	8.0.5

### 3.3 Identification of network performance evaluation metrics

In this study, evaluation metrics commonly used for target detection models, average precision (AP), and mean average precision (mAP) are used to evaluate the model’s performance. The larger the value, the better the performance of the modelis.

Precision (P) is the ratio of the number of correct objects detected to the number of correct objects in the sample, which measures the accuracy of the model detection. Recall (R) is the ratio of the number of correct objects detected to the number of objects in the sample, which measures the percentage of positive samples obtained by the model in the prediction process. Precision and recall can be respectively expressed as:


(13) $$R=\frac{TP}{TP+FN}\times 100\%$$

(14) $$P=\frac{TP}{TP+FP}\times 100\%$$

where TP is the number of correctly identified positive samples, FP is the number of incorrectly identified positive samples, and FN is the number of missed positive samples.

AP refers to the area of the P-R curve plotted by P and R. The area under the curve is the average of all accuracies in different recall values, which measures the accuracy of the model in the defined categories:


(15) $$AP=\sum_{j=0}^{i-1}(R_{\kappa }-R_{\kappa +1})\times P_{\kappa }$$

where *i* is the number of thresholds *j* represents the categories.

mAP refers to the mean value of AP for each category, which measures the accuracy of the trained model on all categories:


(16) $$mAP=\frac{1}{n}\sum_{j=1}^{n}AP_{I}$$

Where *n* represents the total number of categories and *j* represents the class.

### 3.4 Experimental purpose and method

To test the accuracy and efficiency of the multi-cluster green persimmon recognition and detection in complex natural environments based on the improved Faster RCNN developed in this study, backbone network performance optimization experiments and improved Faster RCNN performance detection experiments were conducted.

#### 3.4.1 Backbone network performance optimization experiments

The feature extraction network model is the core part of Faster RCNN, and the good or bad features extracted by the convolutional neural network affect the quality of subsequent training. This paper conducts performance comparison tests between the improved ECA-DetNet network and three classification networks, namely ResNet, AlexNet, and VGGl6, to evaluate the effectiveness of the proposed backbone network improvement.

The same image dataset was used for target recognition and detection experiments. In this study, persimmon fruit recognition is a binary object detection model, so persimmon fruit samples are set as positive samples and the rest of the objects are negative samples. To evaluate the overall effectiveness of the examined classifier, a P-R plot is used to highlight the tradeoff between accuracy and recall.

#### 3.4.2 Improved Faster RCNN performance testing experiment

To evaluate the effectiveness of the improved Faster RCNN method as proposed in this study, its performance is contrasted with the three networks of Faster RCNN, SSD, and yolov3-spp for the identification of green multi-cluster persimmons under different shading degrees, lighting conditions, and shooting intervals.

The experiments replace the feature extraction network of Faster RCNN with ECA-added DetNet and perform multi-scale feature fusion as the final candidate frame region features, while using k-means clustering to determine the anchor frame size. The test set is a randomly selected corresponding image from the entire test set samples, and the test is divided into different occlusion degrees, different illumination, and different shooting intervals as variables.

Target detection with different occlusion degrees: under the condition of controlling the lighting and shooting interval, three groups of heavy, medium, and light occlusion were set up and respectively detected by the three algorithms. Each group was repeated three times, the precision rate, recall rate, and detection time were recorded, and the average of the three trials was recorded as the valid value. The outcomes of the four algorithms are compared to verify if the algorithm improvement proposed in this study can accurately identify the targets under different occlusion situations.

Target detection test under different light conditions: in the complex natural environment of the actual collection, the accuracy of fruit recognition might be affected by issues like exposure on sunny days or low light on cloudy days. The test was set up in three groups: sunny day smooth light, sunny day backlight, and cloudy day backlight. Each group was divided into three types of photos with heavy, medium, and light shading, and the four groups of twelve types of photos were detected by four algorithms. Each group was repeated three times, the average precision (AP) and the mean average precision (mAP) were recorded, and the average of the three times was taken as the valid value. The results of the three algorithms are compared to verify whether the improved algorithm proposed in this study can accurately identify the targets under different lighting conditions.

Target detection test with different shooting intervals: the accuracy of the green persimmon detection model is tested by randomly alternating recognition at different shooting distances, such as near, medium, and far distances. The test is divided into three groups: near, medium, and far distances, with the shooting interval of 10cm~20cm set as near distance, 30cm~50cm as medium distance, and more than 50cm as far distance. Each group was divided into three types of photos: heavy, moderate, and light obscuration. Comparing the results of the four detection algorithms, it has been tested that the improvement of the algorithm used in this study can improve the detection performance of small distant targets.

## 4 Experimental results and analysis

### 4.1 Experimental results of backbone network model performance optimization

**Figure 7:**
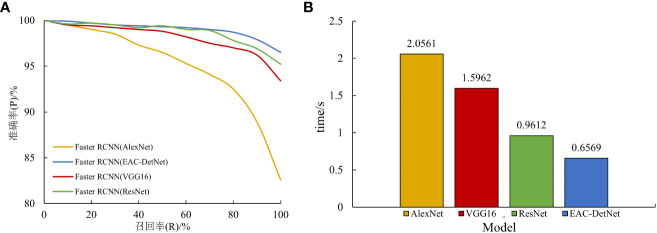
Comparison of P-R plots **(A)** and Detection time **(B)** of different backbone network models.

The P-R plots and detection times of the test set image recognition using four different backbone networks are shown in **Figure 7**.

As can be seen from **Figure 7**, all four algorithms show a decreasing trend in accuracy rate as the recall rate increases. However, the AlexNet classification network exhibits the largest decline, with the accuracy rate close to a linear decline at a recall rate of 80% and an accuracy rate below 85% when the recall rate is 100%. In contrast, the ECA-DetNet algorithm shows a gentle decline, with a more pronounced drop in accuracy only at 80% recall, and the accuracy rate of the network remains above 95% when the recall rate is 100%. The overall P-R plots of the ECA-DetNet algorithm are all higher than those of the ResNet, VGGl6, and AlexNet classification networks, and the change trend is flat, indicating that this study has significantly improved the algorithm’s performance robustness. ECA-DetNet is substantially more accurate than the other three for multiple target fruit recognition, with an accuracy rate of over 95%. The detection time of AlexNet algorithm was 2.06s, which took the longest time, while the detection time of ECA-DetNet algorithm was the shortest with only 0.66s, which was 213%, 142.99%, and 46.32% faster than ResNet, VGGl6, and AlexNet algorithms, respectively. Therefore, ECA-DetNet exhibits significantly better performance than the other three classification networks in terms of detection time, indicating that the algorithm’s detection speed has been considerably improved in this study. To sum up, the effectiveness of the algorithm optimization proposed in this study is demonstrated by the remarkable multifaceted performance of the ECA-DetNet.

### 4.2 Experimental results of improved Faster RCNN performance detection

#### 4.2.1 Detection results of targets with different occlusion degrees

Model training and testing were performed under the same conditions using AP with different occlusion degrees, mAP of combined samples and single image detection time as evaluation metrics. As shown in **Tables 3**, **4**, when the fruits are heavily occluded, the conventional algorithm will miss-detect, and the position of the anchor frame will be shifted substantially. The size of the anchor frame is not adapted to the actual fruit when the fruit is in the independent state in the case of light occlusion. The improved algorithm, on the other hand, is more accurate for fruit recognition and localization. The mAP of the conventional Faster RCNN is 86.6%, whereas for the improved Faster RCNN, it is 98.4%, and the algorithm detection is improved by 11.8% in accuracy, which is significant. The single image detection time of the conventional Faster RCNN is 1.26s, and the single image detection time of improved Faster RCNN is 0.72s, which is a 75% improvement in detection speed. The experiment proves that the improvement of Faster RCNN in this study is obvious and can effectively optimize the training model.

**Table 3:** Actual detection results of the two algorithms.

	Heavy obscuration	Moderate obscuration	Light obscuration
**Conventional Faster RCNN**	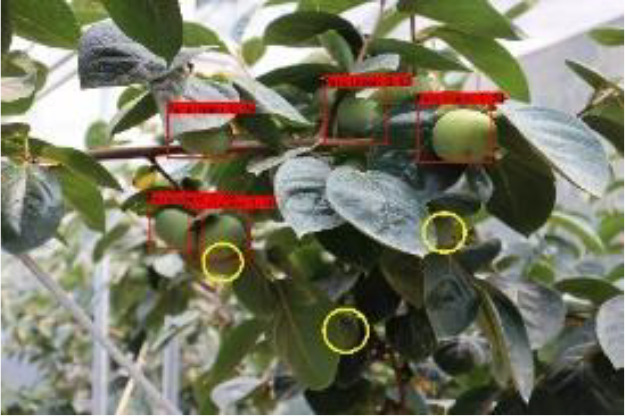	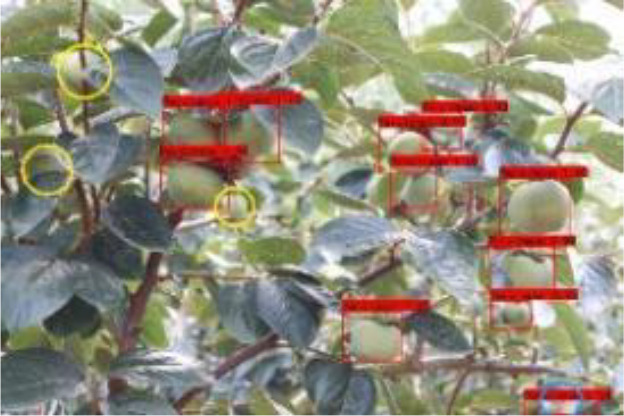	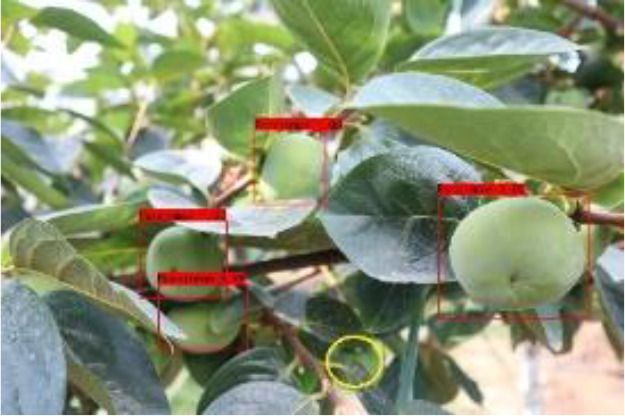
**Improved Faster RCNN**	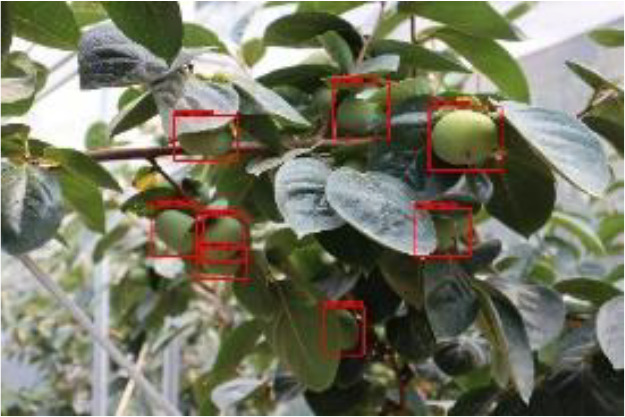	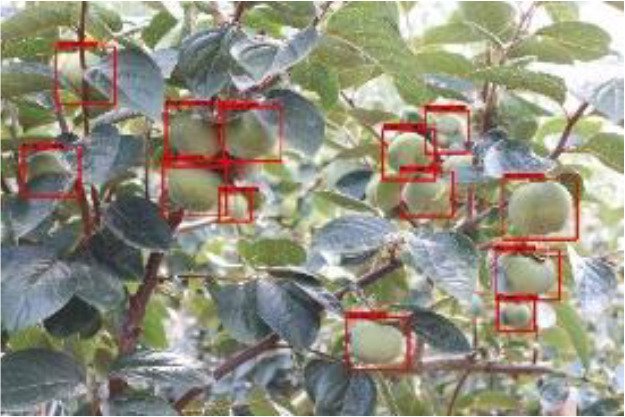	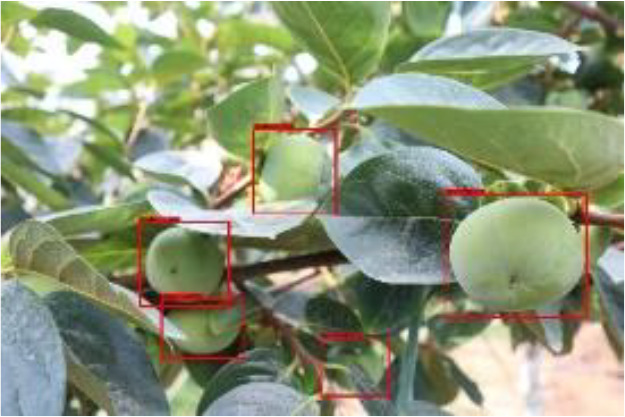

The yellow circular boxes from **Table 3** are the missed fruit markers, and the blue circular boxes are the false fruit markers.

**Table 4:** Comparison of Faster RCNN and improved Faster RCNN algorithms.

Algorithm	AP/%			mAP/%	Single picture detection time/s
	Heavy obscuration	Moderate obscuration	Light obscuration		
Faster RCNN	83.6	87.8	88.3	86.6	1.26
Improved Faster RCNN	97.1	98.6	99.5	98.4	0.72

#### 4.2.2 Detection results of targets with different lighting conditions

As can be seen in **Table 5**, the degree of occlusion increases from left to right, but the improved algorithm compared with the other two can overcome the influence of various lighting to a certain extent. Besides, the superiority of the improved Faster RCNN algorithm recognition accuracy is not obvious, but the small target and backlight target recognition and positioning are more accurate, the advantage is significant.

**Table 5:** Example graphs of some actual detection results of the four algorithms.

	Sunny smooth light	Sunny back light	Cloudy back light
Improved Faster RCNN algorithm	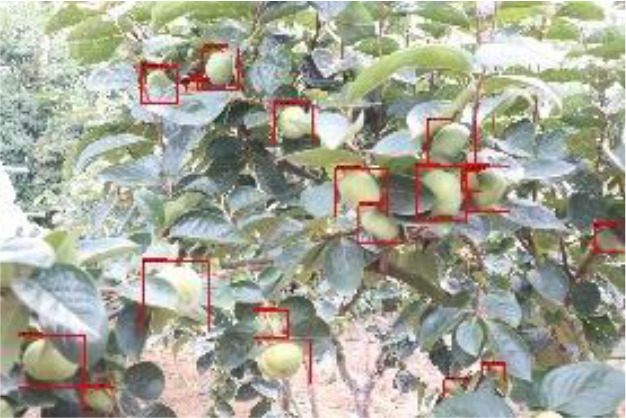	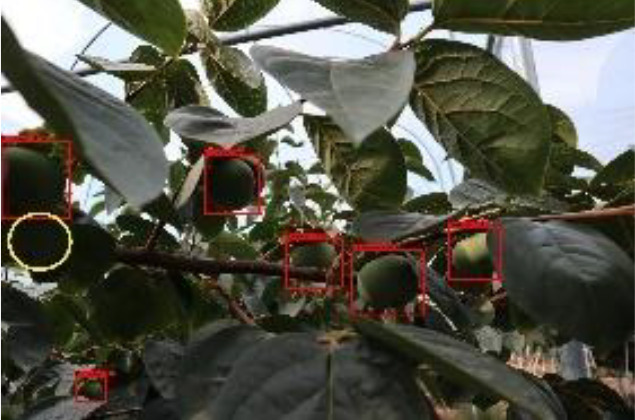	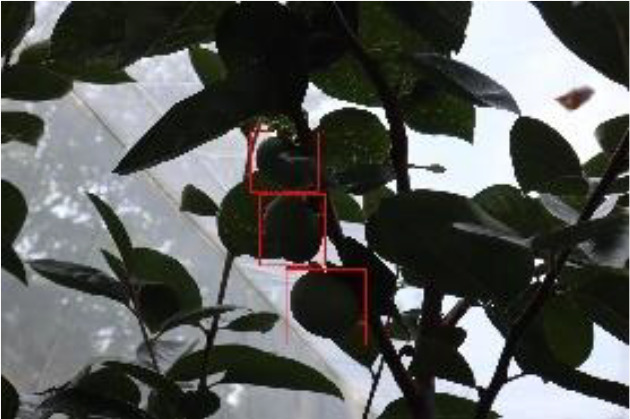
YOLOv7 algorithm	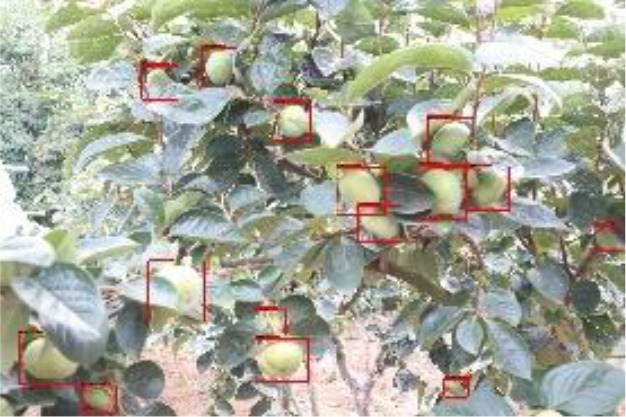	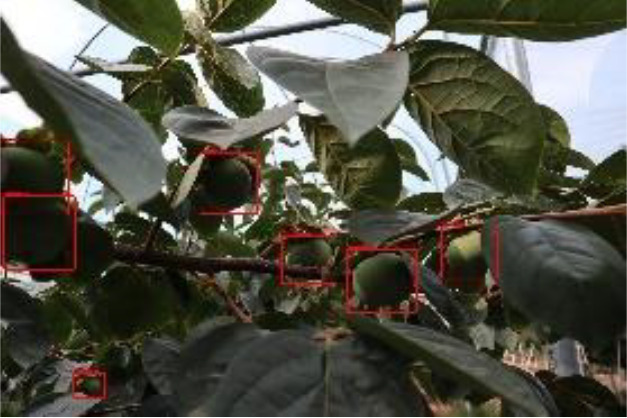	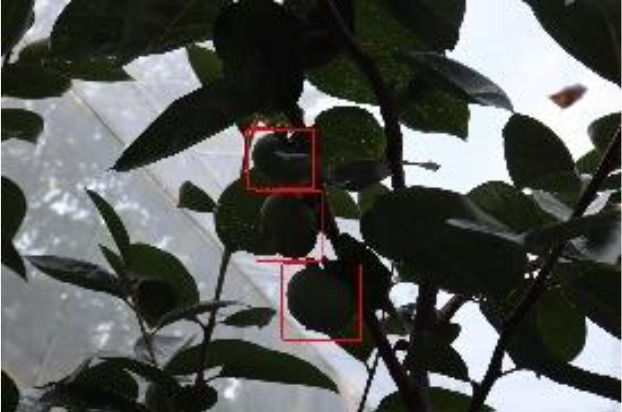
SSD algorithm	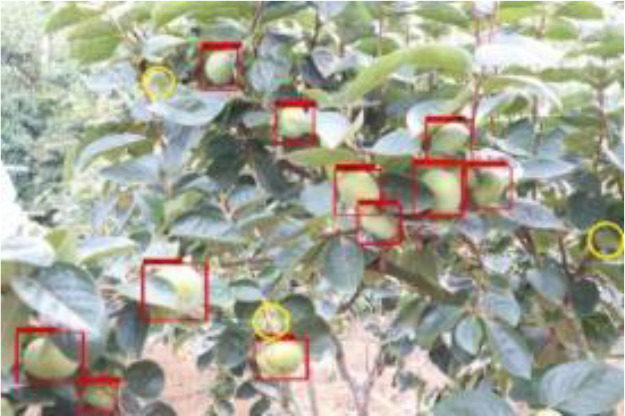	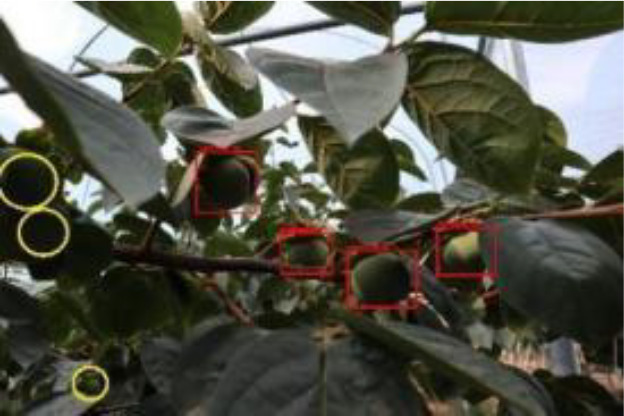	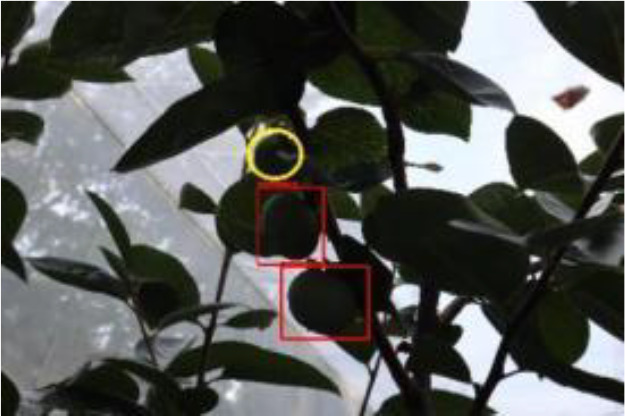
YOLOv3-spp algorithm	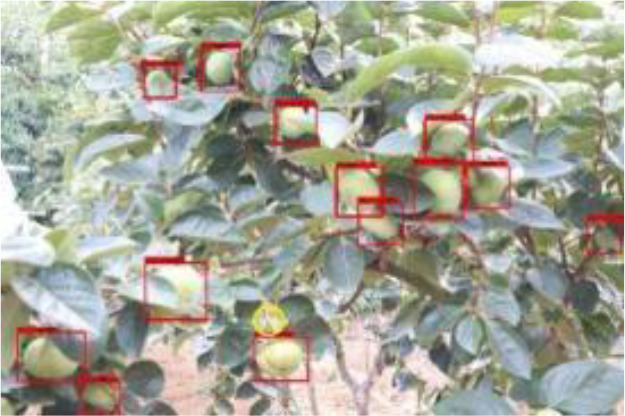	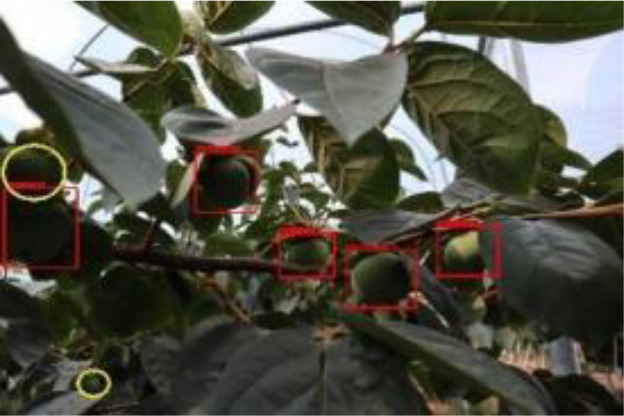	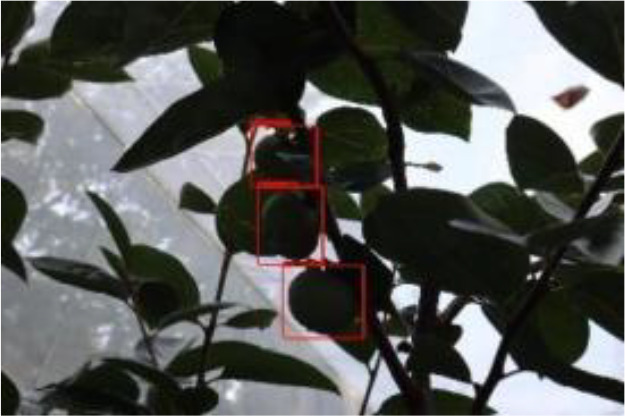

As can be seen from **Table 5**, the difference in detection accuracy of the three target detection algorithms under uniform light conditions is not large, and all can detect the target fruit accurately. However, as the light intensity becomes weaker, the improved Faster RCNN algorithm exhibits outstanding advantages in recognition accuracy. Besides, it is more accurate in recognizing and locating small and backlight targets, which is significantly better than the accuracy of recognition and locating of SSD and YOLOv3-spp algorithms; Compared with the current mainstream algorithm YOLOv7, the detection effect of the two algorithms is comparable under different lighting conditions, and the improved Faster RCNN algorithm can achieve more accurate recognition in some cases of complex light conditions and serious fruit occlusion.

**Figure 8:**
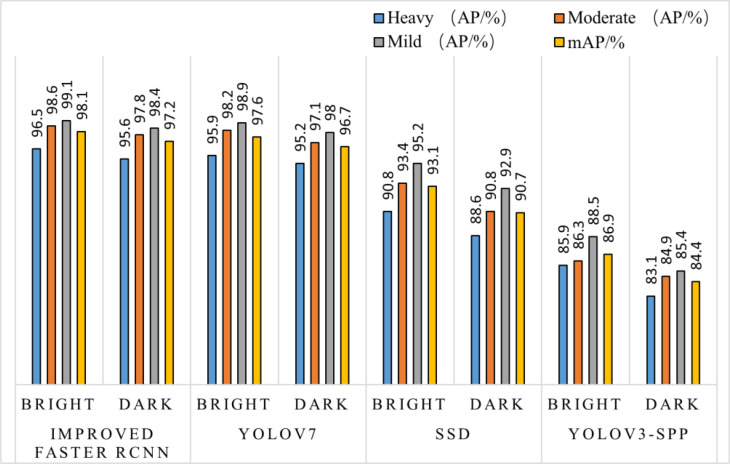
Comparison of four algorithms for detection under different lighting conditions.

The test set samples of sunny and cloudy sky with smooth light and backlight conditions were mixed to increase the robustness of the model, and evaluated with two evaluation indices, AP and mAP, and the results are shown in **Figure 8**. Under different lighting conditions, the overall detection effect level of the improved Faster RCNN algorithm and the YOLOv7 algorithm is not much different; In the case of backlighting without considering the degree of occlusion, the SSD and YOLOv3-spp algorithms show an overall decreasing trend of AP and mAP values compared to the improved algorithm in this paper. The improved algorithm can achieve the highest accuracy value of 98.4% for fruit recognition under this condition, which is an improvement of 5.5%~13% year-on-year. Under the extreme conditions with the highest degree of backlight shading, the AP values of the other two algorithms show a significantly decreasing trend and fluctuate greatly, while the improved algorithm has a decreasing but gentle change within the normal range.

#### 4.2.3 Three different shooting interval target detection results

**Figure 9:**
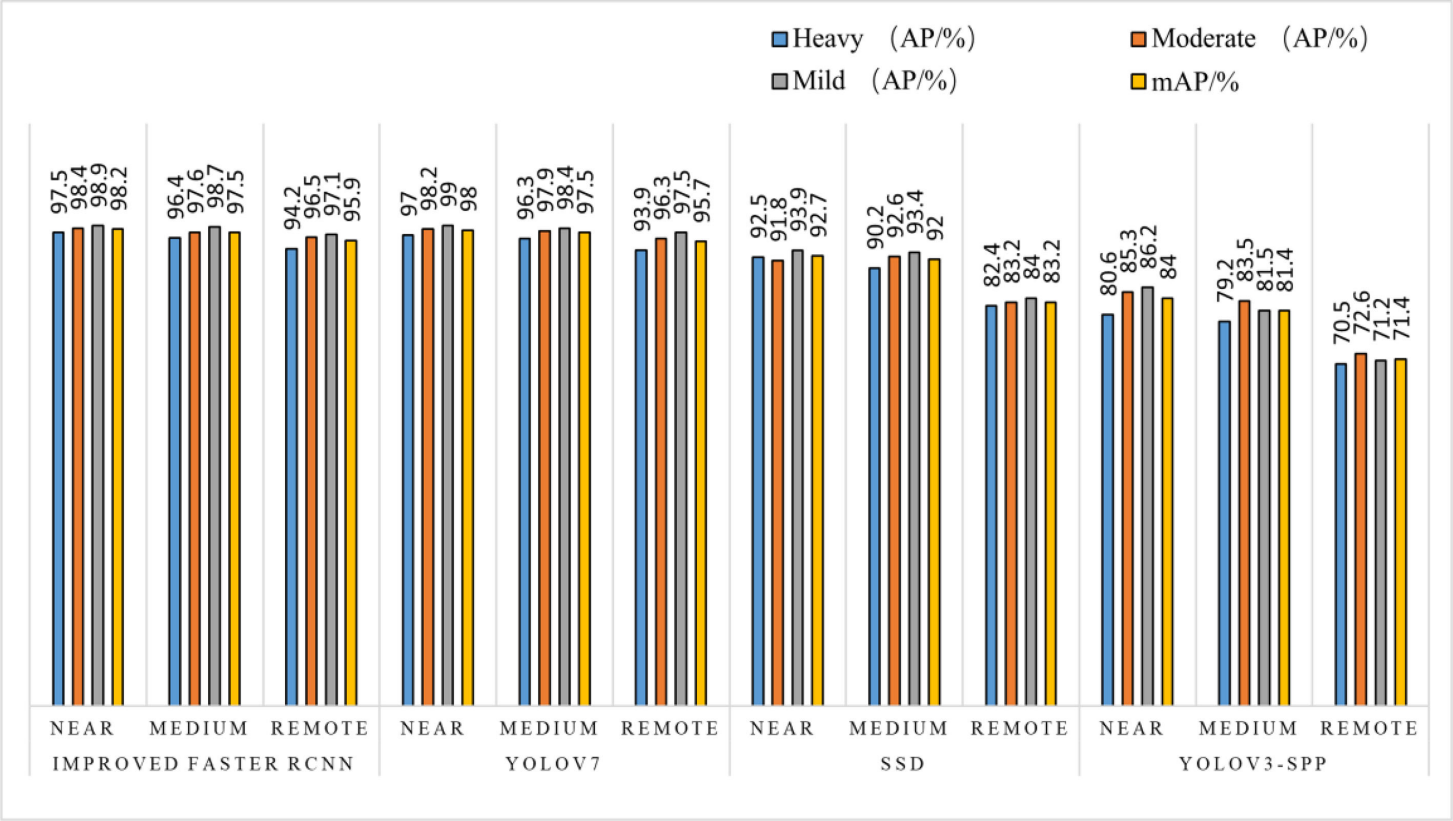
Comparison of the detection of the four algorithms at different shooting distances.

According to the testing of the dataset with different occlusion levels at 3 shooting distances, the detection pairs derived from the three algorithms are shown in **Table 6** and **Figure 9**. **Table 6** demonstrates that the improved Faster RCNN algorithm has a low leakage rate for fruits through the detection result pictures, and it can accurately identify and locate small target objects that are in complex environment in the distant view, which has substantially improved the detection effect compared to the other three algorithms.

**Table 6:** Example graphs of some of the actual detection results of the four algorithms.

	Close proximity	Intermediate distance	Long Distance
Improved Faster RCNN algorithm	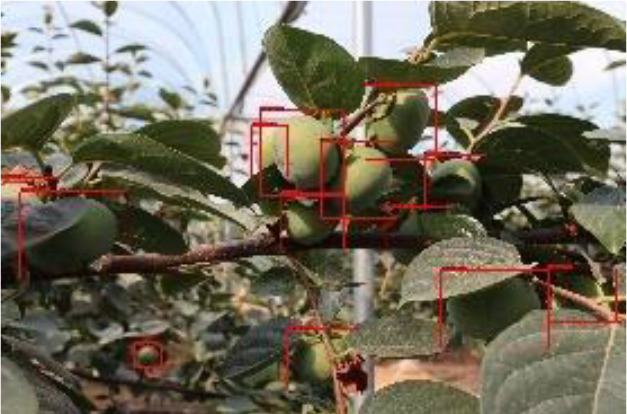	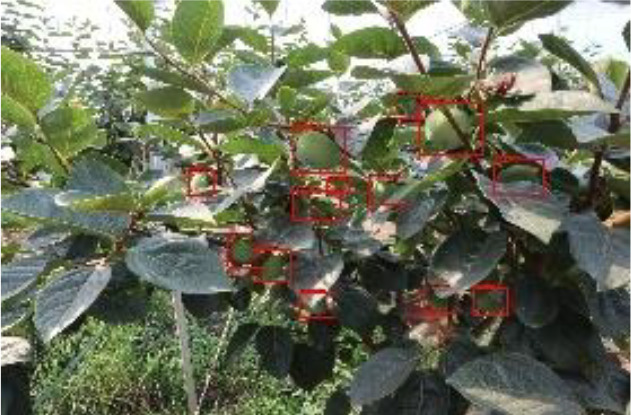	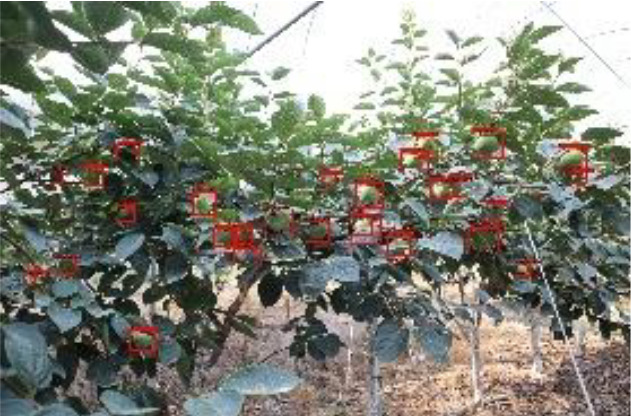
YOLOv7 algorithm	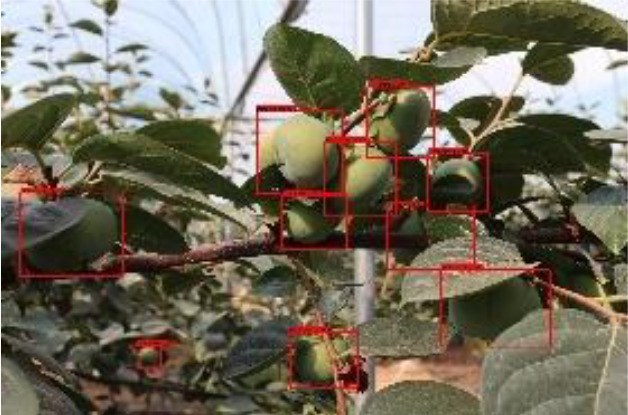	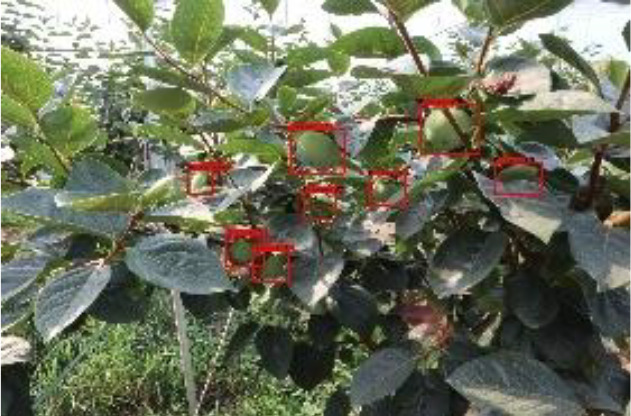	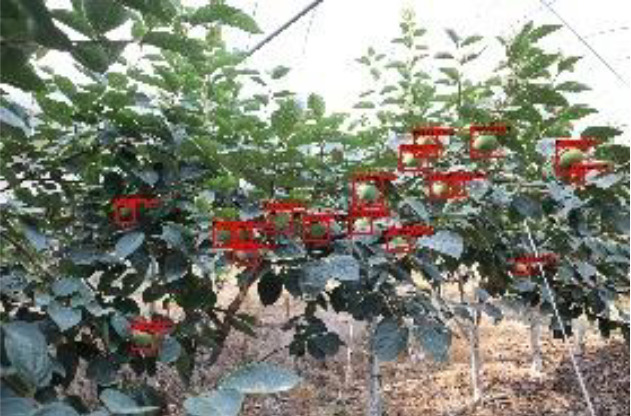
SSD algorithm	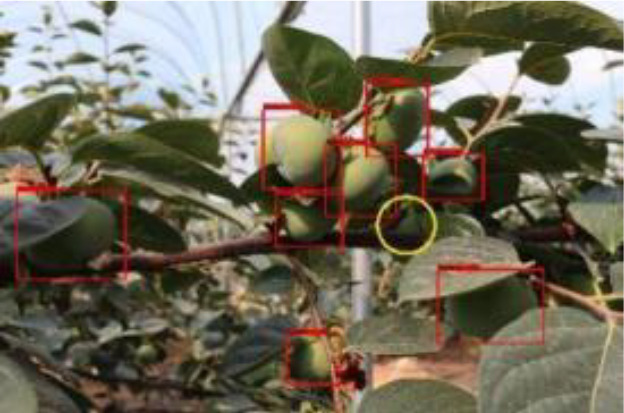	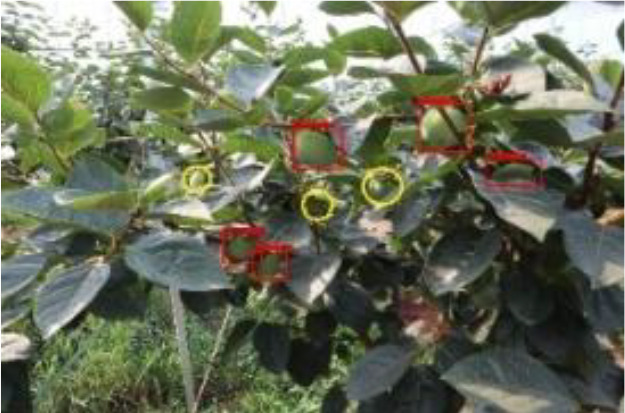	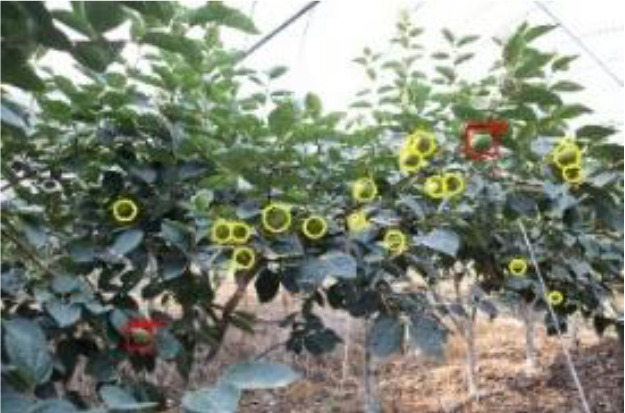
YOLOv3-spp algorithm	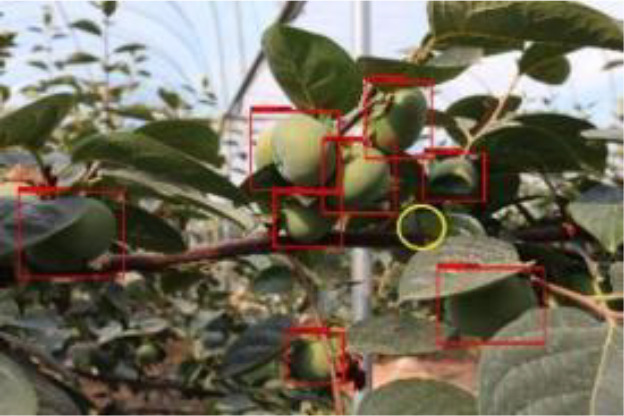	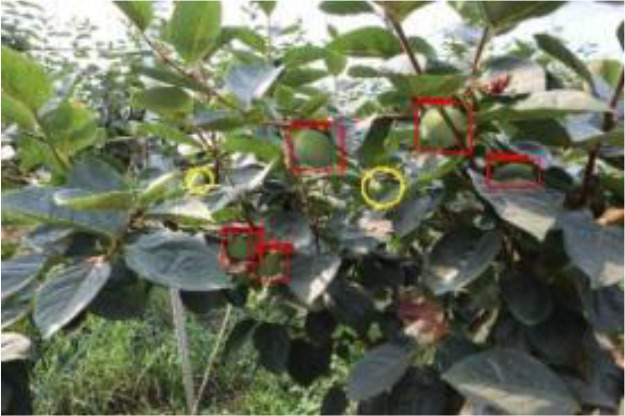	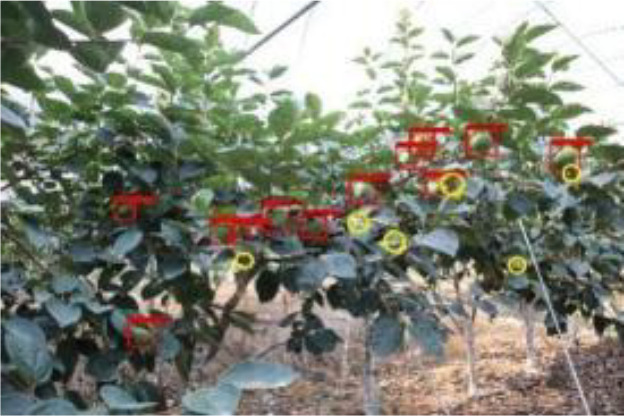

As can be seen in **Figure 9**, in the detection test results of different shooting spacing, the AP values of different occlusion degrees of this paper’s algorithm at different shooting distances have a small variation, while the other three algorithms have relatively up and down variation floating in the same situation. The mAP value of the improved Faster RCNN algorithm can still reach 95.9% in the case of long-distance shooting target objects in heavy occlusion, maintaining a high accuracy, and the comparison tests with other algorithms have improved by 12.7~24.5 percentage points respectively, which is a significant improvement compared to the other two algorithms.

### 4.3 Discussion

It is difficult to detect the target object by the color and outline of the target alone when the target is illuminated by different lights. Based on this issue in different light detection tests, the three target detection algorithms were compared under paralight conditions, and it can be seen in **Figure 8** that while the trend of their target detection results are all on the rise, the AP% value of the improved algorithm under different occlusion conditions changes less, and remains above 96% on average. It can be seen that by adding the weighted channel attention mechanism, the weight of surface feature information and high-level semantic information is enhanced, and useless information is ignored. It allows for improvement in the mAP value of the enhanced algorithm under extreme environmental conditions by 5.0 and 11.2 percentage points, respectively, as compared to the other 2 common target detection algorithms. The improved algorithm has a stronger advantage in recognition and detection under backlight conditions, and the detection is significantly better than the other two algorithms.

After the improved algorithm combines intuitive and abstract features, the multi-scale feature fusion can detect the target object more accurately. Faced with target objects at different shooting distances, the target object to be detected is large for close range shooting and small for long range shooting. Therefore, we must also take into account the accuracy of small target identification in addition to the issue of the target being obscured. The improved algorithm in **Figure 9** improves the overall aspect of AP and mAP values by about 20% compared to the YOLOv3-spp algorithm under this condition of severe occlusion at long distances. By adding attention weights and further multi-scale fusion of the extracted features, this improved feature extraction increases the accuracy of fruit recognition to a large extent compared to the simple feature extraction of YOLOv3-spp. Therefore, the improved algorithm effectively solves these two problems, and **Figure 8**, **Figure 9** illustrate that the improved algorithm can be stably applied to green persimmon recognition detection in different scenarios. Meanwhile, the improved weighted ECA-DetNet is clearly applicable to small target detection, and the multi-scale feature fusion can effectively detect the occluded targets. The schematic diagram of the detection results before and after the improved algorithm in **Table 3** shows that in the experiments with different degrees of occlusion, the improved Faster RCNN algorithm has a low miss detection rate and error detection rate; the anchor frame position size is adapted to the target object in the picture. In the complex orchard environment, the improved algorithm in this paper has a similar detection effect on green multi-tufted persimmons with the current mainstream algorithm YOLOv7.

Early fusion is used in the Faster RCNN algorithm to enhance the utilization of information from different feature layers, i.e., multi-layer features are fused first, and the predictor is trained on the fused features afterwards. This approach can effectively cope with the problems faced by target detection of obscured objects in complex environments with near views. After improving the algorithm, the computational effort brought on by different dimensions is solved in advance in the multi-scale feature fusion so that the fused feature layers have the same number of channels and are directly connected afterwards to improve the detection accuracy by the complementarity of features in different layers. The anchor frame size is re-determined using k-means clustering method to make it closer to the actual edge of the target. The accuracy of target detection is likewise increased in regression prediction, and the enhanced algorithm in this study improves detection accuracy while also ensuring detection speed. It is expected to be applied to persimmon fruit growth information monitoring in the future, and also provides technical support for subsequent automated harvesting.

## 5 Conclusion

In this paper, we design a green persimmon recognition model with good robustness and accuracy against background interference, fruit occlusion, and other factors by improving the Faster RCNN algorithm, which resolves issues like difficulty in recognizing near scenery occlusion in complex environment. By replacing the DetNet backbone feature extraction network, adding ECA attention mechanism, and adjusting and optimizing the model’s structure and parameters, the feature layers of different depths are combined with multi-scale features to improve the performance of algorithm retrieval robustness, speed, and accuracy.

Through backbone network performance optimization experiments, the ECA-DetNet algorithm refined in this study has been significantly improved in terms of speed, accuracy, and robustness, which proves the effectiveness of this study on algorithm optimization. The improved Faster RCNN performance detection test is compared with the conventional Faster RCNN, SSD, and yolov3-spp target algorithms from multiple perspectives of different occlusion degree, different illumination, and different shooting intervals. The experimental findings demonstrate that the improved Faster RCNN has significantly better detection speed and accuracy compared to other algorithms for heavily occluded targets, targets with backlight conditions on cloudy days, and small targets at long distances. The effectiveness of the algorithm optimization in this study is demonstrated, and the mAP value of the improved Faster RCNN algorithm has improved to 11.8 percentage points compared to the Faster R-CNN. The near-view, complex environment interference resistant multi-cluster green persimmon detection and identification algorithm in this study can rapidly identify immature green persimmons in natural environments and enable growth state monitoring, which provides effective technical support for the development of smart orchards. The method proposed in this study can be applied to the detection of green fruits in unstructured orchard environment, it can be applied to the detection of green fruits in unstructured orchard environment, not only limited to the identification research of persimmons, but also applied to the information monitoring of different green growth periods of fruits, and at the same time provides theoretical and technical support for the subsequent visual system of automatic inspection and picking mechanization of orchard operations, and provides effective technical support for the intelligent and digital development of smart orchards.

## Data availability statement

The datasets presented in this article are not readily available because privately owned. Requests to access the datasets should be directed to m17634935951@163.com.

## Author contributions

LY: conceptualization, methodology, supervision, funding acquisition, and project administration. RH: methodology, software, validation, investigation, and writing - original draft. ZZ: investigation, formal analysis, and writing - review & editing. MF: supervision, visualization, and project administration. ZP: data curation. WL: validation. FR: resources. All authors contributed to the article and approved the submitted version.
